# VLA-MP: A Vision-Language-Action Framework for Multimodal Perception and Physics-Constrained Action Generation in Autonomous Driving

**DOI:** 10.3390/s25196163

**Published:** 2025-10-05

**Authors:** Maoning Ge, Kento Ohtani, Yingjie Niu, Yuxiao Zhang, Kazuya Takeda

**Affiliations:** 1 Graduate School of Informatics, Nagoya University, Furo-cho, Chikusa-Ward, Nagoya 464-8601, Japan; 2 RoboSense Technology Co., Ltd., 701 Block B, 800 Naxian Road, Pudong, Shanghai 200131, China; 3 Tier IV Inc., Jacom Building, 1-12-10 Kitashinagawa Shinagawa-ku, Tokyo 140-0001, Japan

**Keywords:** Vision-Language-Action models, multimodal perception, autonomous driving, large language models, trajectory planning

## Abstract

Autonomous driving in complex real-world environments requires robust perception, reasoning, and physically feasible planning, which remain challenging for current end-to-end approaches. This paper introduces VLA-MP, a unified vision-language-action framework that integrates multimodal Bird’s-Eye View (BEV) perception, vision-language alignment, and a GRU-bicycle dynamics cascade adapter for physics-informed action generation. The system constructs structured environmental representations from RGB images and LiDAR, aligns scene features with natural language instructions through a cross-modal projector and large language model, and converts high-level semantic hidden states outputs into executable and physically consistent trajectories. Experiments on the LMDrive dataset and CARLA simulator demonstrate that VLA-MP achieves high performance across the LangAuto benchmark series, with best driving scores of 44.3, 63.5, and 78.4 on LangAuto, LangAuto-Short, and LangAuto-Tiny, respectively, while maintaining high infraction scores of 0.89–0.95, outperforming recent VLA methods such as LMDrive and AD-H. Visualization and video results further validate the framework’s ability to follow complex language-conditioned instructions, adapt to dynamic environments, and prioritize safety. These findings highlight the potential of combining multimodal perception, language reasoning, and physics-aware adapters for robust and interpretable autonomous driving.

## 1. Introduction

In complex and dynamic real-world driving environments, human drivers can effortlessly handle various emergencies and rare corner cases by leveraging their rich world knowledge and powerful reasoning capabilities. However, current autonomous driving systems often demonstrate inadequate performance when facing these challenges. Although deep learning-based end-to-end autonomous driving methods have achieved remarkable results in standard benchmark tests, they frequently lack deep understanding of complex scenarios and flexible reasoning capabilities [1,2].

**Figure 1:**
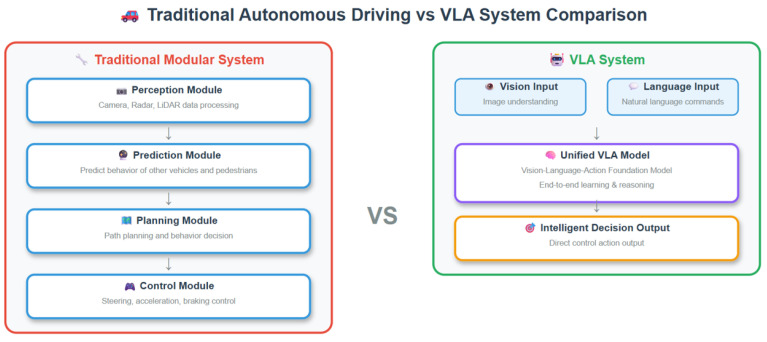
Comparison between traditional autonomous driving systems vs. VLA driving systems.

The emergence of vision-language-action (VLA) models brings new promise to this challenge [3]. As shown in Figure 1, unlike traditional modular autonomous driving systems, VLA models break down the boundaries between perception, prediction, planning, and control modules, directly generating intelligent driving decisions from multimodal inputs (vision and language) through a unified framework. By integrating world knowledge from large-scale pre-trained vision-language models with domain-specific autonomous driving expertise, VLA models can deeply understand complex driving contexts and natural language instructions like human drivers, directly generating corresponding intelligent driving behaviors [4,5].

Recent research demonstrates that VLA models have achieved significant progress in multitask joint training and complex scene reasoning, redefining the capability boundaries of autonomous driving systems. These models can not only handle traditional perception and planning tasks but also demonstrate promising capabilities in understanding complex driving scenarios and executing language-conditioned driving behaviors [6,7,8,9,10,11,12,13,14].

In recent years, breakthroughs in several key technologies have laid the foundation for VLA autonomous driving systems [4]. Multimodal perception and BEV fusion technology has evolved from simple sensor fusion to deep semantic understanding, where Bird’s Eye View (BEV) representation successfully unifies the processing pipeline for RGB camera and LiDAR point cloud data [15,16,17,18,19]. The introduction of Transformer architecture has further enhanced sequence modeling and attention mechanism capabilities, enabling systems to better process spatiotemporal information [20,21,22,23]. Meanwhile, the powerful reasoning capabilities demonstrated by large language models enable systems to understand complex traffic rules and driving instructions, providing new possibilities for intelligent driving decision making [24].

Despite the maturing technological foundation, practical application of VLA autonomous driving systems still faces critical challenges [4]. The core issue lies in effectively fusing multimodal feature representations [25]. Traditional feature concatenation methods struggle to establish deep cross-modal semantic associations, while existing semantic-to-action mapping methods often lack consideration of vehicle physical characteristics, resulting in feasibility issues in generated trajectories during actual execution [2]. Additionally, maintaining language understanding capabilities while achieving multitask collaborative optimization remains an urgent problem in system design [26].

To address these challenges, this paper proposes a closed-loop VLA-based autonomous driving training framework that deeply integrates multimodal perception, language understanding, and physical constraint modeling for end-to-end intelligent driving decisions. The main contributions of this work are as follows:

1. We propose a unified end-to-end VLA framework that integrates multimodal BEV perception, vision-language understanding, and physically constrained action generation, achieving seamless mapping from sensor observations and natural language instructions to executable control commands, with demonstrated closed-loop operation and competitive driving performance in the CARLA simulation environment.
2. We extract hierarchical map, scene, and trajectory features from BEV space, characterizing static road topology, dynamic traffic participants, and future motion trends, respectively, and pass these features to the large language model to bridge perception and cognition.
3. We design a GRU-bicycle model cascade adapter where the GRU processes temporal semantic information and the bicycle model ensures trajectory compliance with vehicle dynamics constraints, guaranteeing physical feasibility and executability of generated trajectories.
4. We develop a three-stage progressive training strategy encompassing environmental perception pre-training, vision-language alignment, and end-to-end fine-tuning, enabling effective knowledge transfer and stable convergence in the complex multimodal learning process.

## 2. Related Works

### 2.1. End-to-End Autonomous Driving

End-to-end autonomous driving represents a paradigm shift from traditional modular approaches, directly mapping sensor inputs to driving actions through a unified neural architecture, thereby avoiding information loss between modules in traditional pipelines. The unified perception-prediction-planning framework has become the cornerstone of this field. UniAD [23] introduced a comprehensive framework that integrates multiple driving tasks, such as object detection, tracking, motion prediction, occupancy prediction, and planning, into a single neural network, demonstrating the performance advantages of cross-task joint optimization over independent modular methods. Building on this foundation, several methods have adopted vectorized representations and sparse attention mechanisms to efficiently process complex traffic scenarios in a unified vector space and achieve direct mapping from perception to planning through spatiotemporal feature learning [27,28,29,30], further highlighting the advantages of end-to-end multimodal fusion and joint optimization, including improved safety and performance.

The deployment challenges of end-to-end systems primarily lie in model complexity and generalization capabilities. Several works have identified key issues in handling long-tail scenarios and proposed methods to improve generalization and domain adaptation through synthetic data augmentation and transfer learning techniques [31,32], laying an important foundation for the practical application of end-to-end autonomous driving.

Although end-to-end methods exhibit advantages in unified optimization, existing systems are primarily confined to traditional perception-prediction-planning paradigms and lack the ability to understand and reason about natural language instructions. These systems are unable to handle complex driving scenarios that require commonsense reasoning and language interaction, making it difficult to achieve truly human-machine interactive intelligent driving, thus posing an urgent need for the development of unified vision-language-action frameworks.

### 2.2. Multimodal Perception and BEV Fusion

Integrating multiple sensor modalities through Bird’s Eye View (BEV) representations has revolutionized autonomous driving perception, providing a unified framework for handling heterogeneous sensor data while maintaining spatial consistency. RGB-LiDAR fusion methods have evolved from simple concatenation to complex cross-modal learning frameworks. TransFusion [33] introduced a transformer-based fusion architecture that learns attention weights across different modalities, enabling adaptive sensor integration based on environmental conditions. Several subsequent methods have further promoted bidirectional information flow between RGB and LiDAR features and bridged semantic gaps under adverse conditions [34,35], enhancing perception robustness.

Unified BEV representation learning has become the dominant paradigm for multimodal fusion. BEVFormer [17] laid the foundation by demonstrating how transformer architectures can effectively learn BEV representations from multi-camera inputs through spatial cross-attention mechanisms. BEVFusion [16] extended this concept by integrating LiDAR point clouds, showing significant performance improvements in detection and segmentation tasks. Recent advances include addressing depth estimation challenges in camera-based BEV learning and introducing position-guided attention mechanisms for more accurate 3D object detection in BEV space [19,36].

Although existing multimodal fusion methods have made significant progress in perception tasks, deficiencies remain in feature representation and cross-modal alignment. Existing methods primarily employ simple feature concatenation or attention mechanisms for modal fusion, lacking hierarchical feature extraction strategies to distinguish different semantic levels, such as static environmental structures, dynamic traffic participants, and future motion trends.

### 2.3. Vision–Language Models in Autonomous Driving

Integrating vision–language models into autonomous driving systems represents a major leap toward human-like reasoning and decision-making capabilities, enabling vehicles to understand and respond to natural language instructions while maintaining comprehensive scene perception. Large language models for scene understanding and decision making have demonstrated exceptional capabilities in handling complex driving scenarios. LLM4Drive [1] provides a comprehensive review on adapting large language models to autonomous driving tasks, highlighting their potential in managing complex reasoning and commonsense understanding. GPT-Driver [13] demonstrates how generative pre-trained transformers can be fine-tuned for driving-specific tasks, including scene description, risk assessment, and action planning.

Interpretable driving behavior generation is becoming increasingly important for building trust and ensuring system safety. DriveGPT4 [11] pioneered the integration of large language models into interpretable driving decisions, showing how natural language explanations can accompany driving behaviors. LMDrive [12] extends this concept by incorporating chain-of-thought reasoning, enabling the system to express decision processes step by step. ADAPT [37] further enhances interpretability by introducing adaptive reasoning mechanisms that can provide explanations at varying levels of detail based on scene complexity and user needs.

Instruction-following driving systems represent a key step toward more interactive and adaptive autonomous driving vehicles. Talk2Drive [38] extends this concept by enabling real-time dialogue between passengers and the driving system, facilitating dynamic route changes and driving style adjustments. NuPrompt [39] introduces a prompting framework capable of handling complex multi-step instructions.

However, existing VLA methods exhibit key deficiencies in the physical feasibility of trajectory generation. Most methods directly output trajectory coordinates from language models, lacking modeling of vehicle dynamics constraints, resulting in trajectories that may violate physical laws and cannot be executed in practice. Additionally, existing semantic-action mapping mechanisms often overlook the processing of temporal dynamic information, making it difficult to fully utilize historical state information for continuous trajectory planning. These issues underscore the importance of integrating vehicle physical models and temporal state modeling in VLA systems.

## 3. Methodology

This paper proposes VLA-MP (Multimodal Perception and Physics-Informed Action Generation), an end-to-end vision-language-action framework that integrates multimodal BEV perception, vision-language bridging, and physics-constrained action generation as its core components, achieving a unified mapping from sensor observations and natural language instructions to executable control commands. Compared with traditional modular systems, VLA-MP can fuse scene semantics with language priors within a single architecture, thereby enhancing understanding and reasoning capabilities in complex scenarios.

**Figure 2:**
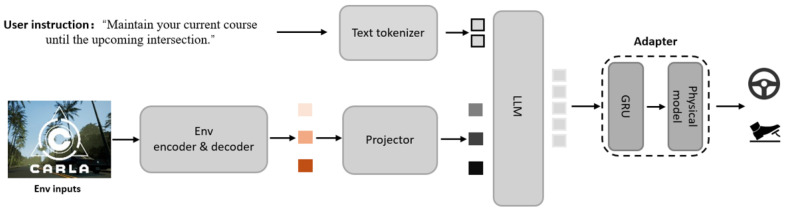
Overview of the proposed VLA-MP autonomous driving framework. The system integrates pre-trained env encoder and decoder components, a driving VLA module, and an adapter to achieve end-to-end mapping from multimodal inputs to control commands. The Env encoder and decoder components are pre-trained from the multimodal environmental perception module (detailed in Figure 3).

**Figure 3:**
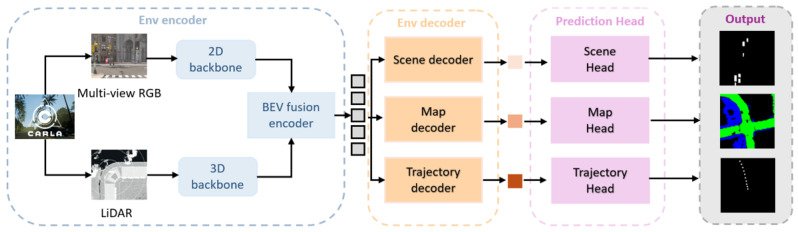
Multimodal Environmental Perception Module used in Stage 1 pre-training. The module processes RGB images and LiDAR data through separate 2D and 3D backbones, fuses them via a BEV fusion encoder, and extracts hierarchical features through scene, map, and trajectory decoders with corresponding prediction heads for multitask learning.

As shown in Figure 2, the system integrates pre-trained environmental components, a driving VLA module, and an adapter for end-to-end processing. The system takes multi-view RGB images, LiDAR point clouds, and user instructions as inputs. The Env encoder and decoder components, which are pre-trained from the multimodal environmental perception module (Figure 3), construct structured environmental representations in BEV space covering dynamic traffic participants, static road semantics, and motion priors. These feature outputs are then fed into the driving VLA module to perform vision-language fusion and generate decisions.

**Figure 4:**
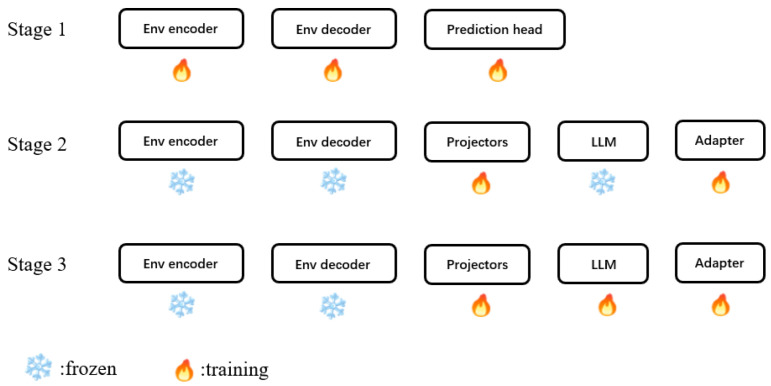
Three-stage training strategy. Stage 1 performs multimodal environmental perception pre-training, Stage 2 conducts vision–language alignment training, and Stage 3 performs end-to-end fine-tuning.

As illustrated in Figure 4, training adopts a three-stage strategy: Stage 1 performs pre-training of the multimodal environmental perception module components (Env encoder, Env decoder, and Prediction heads), focusing on multitask joint optimization of perception and environmental modeling to obtain robust and reusable BEV representations; Stage 2 freezes the pre-trained Env encoder and decoder from the environmental perception module, text tokenizer, and LLM, training only the projectors and adapter, where the Env encoder and decoder output feature-level representations (discarding prediction heads) to learn the alignment mapping from BEV features to language space; and Stage 3 freezes the pre-trained Env encoder and decoder from the environmental perception module and text tokenizer, jointly training the projectors, LLM, and adapter to further optimize multimodal reasoning and instruction-conditioned driving decision generation capabilities. Finally, the adapter converts high-level semantic hidden states output by the LLM into executable trajectories and low-level control commands (steering, throttle, brake) that satisfy vehicle dynamics constraints. This design deeply couples the structured world model provided by BEV with the knowledge and reasoning capabilities of LLMs, providing a robust, interpretable, and executable decision foundation for complex, dynamic, and language-conditioned driving tasks.

### 3.1. Multimodal Environmental Perception Module

As shown in Figure 3, the multimodal environmental perception module serves as the foundational component for Stage 1 pre-training, responsible for unifying heterogeneous data from diverse sensors into structured BEV representations and extracting hierarchical features including scene semantics, road topology, and motion priors through specialized environmental understanding heads. During VLA-MP training, we utilize the pre-trained env encoder and decoder components from this module. This module employs a Transformer-based fusion architecture [30,40] that maps RGB images and LiDAR point cloud data into a unified BEV grid space, providing stable and rich environmental representations for subsequent language-vision alignment and trajectory generation.

The system first processes RGB images and LiDAR point cloud data through separate 2D and 3D backbone networks. For multi-view RGB inputs, ResNet50 [41] is employed as the 2D backbone to extract deep features from each viewpoint, with learnable view embeddings and positional encodings to distinguish spatial relationships between different cameras. LiDAR data is converted to pseudo-image representation through the PointPillar [42] network and subsequently processed by a 3D convolutional backbone to extract geometric structural features. Features from both modalities are then deeply fused in the BEV fusion encoder through cross-modal attention mechanisms [12,17]. Specifically, RGB features from each viewpoint are processed through convolutional projection and then flattened into a sequence format for the Transformer encoder with multi-head self-attention to fuse multi-view information. LiDAR features processed by PointPillar serve as BEV queries in a H×W format. The BEV decoder then uses these LiDAR-derived queries to attend to the encoded RGB features through cross-attention, where the RGB tokens serve as keys and values, while LiDAR features serve as queries, enabling the model to selectively extract relevant visual information for each BEV grid location. This process generates a unified BEV feature representation $F_{\mathrm{BEV}}\in R^{B\times D\times H\times W}$, where *B* denotes batch size, *D* represents feature dimension, and *H*, *W* indicate BEV grid resolution.

To support multitask learning and hierarchical environmental understanding, the system incorporates three specialized environmental understanding heads, each equipped with task-specific query mechanisms and network architectures to extract corresponding semantic information. The scene head interacts with BEV features through learnable query tokens $Q_{scene}$ and utilizing multi-layer linear transformation networks [43] to output 8-dimensional feature vectors containing target confidence, center point coordinates, bounding box dimensions, heading angle, and motion velocity, achieving precise detection and state estimation for traffic participants including vehicles, pedestrians, and bicycles. The map head employs an upsampling decoder structure [44], decoding from BEV features through specialized query tokens $Q_{map}$ and progressively upsampling to high resolution, generating three-channel semantic maps containing road markings, drivable lanes, and sidewalks to provide precise road topology information for path planning. The trajectory head utilizes a GRU temporal predictor structure [45], extracting temporally correlated feature representations through query tokens $Q_{trajectory}$ and modeling temporal dependencies of vehicle motion using GRUs, providing prior information containing motion trends and scene constraints for the subsequent physics-constrained trajectory generation module.

Through this hierarchical environmental understanding design, the system can simultaneously process static scene structures and dynamic traffic situations, providing comprehensive and structured environmental cognitive foundations for subsequent language-conditioned reasoning and action generation. The scene head employs threat-aware multitask detection loss [30] for supervised learning:

(1) $$L_{scene}=\lambda_{prob}L_{prob}+\lambda_{loc}L_{loc}+\lambda_{box}L_{box}+\lambda_{ori}L_{ori}$$

where $L_{prob}$ is the probability loss using binary cross-entropy to supervise object existence prediction; $L_{loc}$ is the location loss employing L1 distance to supervise center point coordinate prediction; $L_{box}$ is the bounding box loss using L1 distance to supervise object dimension prediction; and $L_{ori}$ is the orientation loss using L1 distance to supervise heading angle estimation. All regression losses employ dynamic threat-aware weighting coefficients. These four loss components are essential for comprehensive object detection: probability loss ensures accurate object identification, location and box losses provide precise spatial localization needed for safe trajectory planning, and orientation loss captures vehicle heading information critical for predicting future motion patterns and potential collision risks. The map head utilizes binary cross-entropy loss for pixel-wise semantic segmentation supervision:

(2) $$L_{map}=-\sum_{h,w}\left[m_{h,w}\log\sigma \left({\hat{m}}_{h,w}\right)+\left(1-m_{h,w}\right)\log\left(1-\sigma \left({\hat{m}}_{h,w}\right)\right)\right]$$

where $m_{h,w}$ are ground truth semantic labels and ${\hat{m}}_{h,w}$ are predicted semantic maps at spatial coordinates (h,w). The trajectory head adopts L1 loss for trajectory prediction supervision:

(3) $$L_{traj}=\sum_{t=1}^{T}\left|\right|p_{t}-{\hat{p}}_{t}{\left|\right|}_{1}$$

where $p_{t}$ and ${\hat{p}}_{t}$ represent ground truth and predicted trajectory coordinates at time step *t*, respectively, and *T* is the prediction horizon.

### 3.2. Vision–Language Bridge and Large Language Action Model

The vision–language bridge module serves as the critical component connecting BEV environmental perception with intelligent decision making, responsible for precisely aligning feature representations from the environmental perception module with natural language instructions and achieving instruction-conditioned scene understanding and high-level decision generation through the large language action model. As illustrated in the driving VLA module of Figure 2, this module primarily consists of cross-modal projectors and a large language model (LLM), realizing unified processing of multimodal information by mapping BEV features to language space and fusing them with text tokens. The complete vision–language bridging process is summarized in Algorithm 1.
**Algorithm 1** Vision–Language Bridge and Cross-Modal Alignment **Require:**BEV decoder features $\left\{F_{\mathrm{scene}},F_{\mathrm{map}},F_{\mathrm{traj}}\right\}$; instruction *T*
 
**Ensure :**waypoints
 
// Cross-Modal Feature Projection

**_1_** Fuse environmental features: $F_{\mathrm{features}}\leftarrow \mathrm{Concat}\left(F_{\mathrm{scene}},F_{\mathrm{map}},F_{\mathrm{traj}}\right)$;

**_2_** Initialize learnable query tokens Q;

**_3_** Cross-attention interaction: $Z_{\mathrm{visual}}\leftarrow \mathrm{QFormer}\left(Q,F_{\mathrm{features}}\right)$;

**_4_** Project to LLM space: $V\leftarrow \mathrm{LinearProj}(Z_{\mathrm{visual}})$;
 
// Multimodal Token Fusion

**_5_** Text embedding: $E_{\mathrm{text}}\leftarrow \mathrm{Embed}\left(\mathrm{Tokenizer}\left(T\right)\right)$;

**_6_** Token concatenation: $E_{\mathrm{joint}}\leftarrow \mathrm{Concat}\left(E_{\mathrm{text}},V\right)$;
 
// LLM Reasoning and Decision Generation

**_7_**
$R\leftarrow {\mathrm{LLM}}_{\mathrm{hidden}}\left(E_{\mathrm{joint}}\right)$;

**_8_** waypoints ← WaypointsDecoder(R);

The cross-modal projector employs a BLIP2-like [46] Q-Former architecture to achieve vision–language alignment. Unlike conventional cross-attention that directly maps features, Q-Former uses a fixed set of learnable query tokens to selectively extract task-relevant information from BEV features. The system performs cross-attention interaction between learnable query tokens and BEV features to extract key semantic information, which is then mapped to the large language model’s feature space through linear projection layers. During Stage 2 training, the pre-trained environmental components discard prediction heads and output only feature-level representations from the environmental decoder, containing semantic information from scene understanding, map generation, and trajectory planning branches. The Q-Former selectively extracts driving-task-relevant key information from these BEV features through cross-attention mechanisms, generating compact visual representations. Subsequently, linear projection layers ensure precise alignment between visual semantics and language semantics at the feature level, ultimately outputting visual token representations.

The large language action model employs pre-trained language models based on LLaVA [47] or Qwen [48] architectures as the reasoning backbone, achieving instruction-conditioned driving decision generation by processing joint sequences of text tokens and visual tokens. The system processes pre-existing natural language driving instructions from the LMDrive dataset rather than generating new command prompts, converting these given instructions into text token sequences through a text tokenizer, then concatenates them with visual tokens processed by the projector to form unified multimodal input sequences. The large language model leverages its powerful self-attention mechanism to simultaneously model complex dependencies between text instructions and visual scene information, performing deep understanding of complex driving scenarios through commonsense knowledge and reasoning capabilities acquired during pre-training. The model can handle various types of driving instructions, such as “Maintain your current course until the upcoming intersection,” and generate corresponding high-level semantic decisions based on current visual observations.

Through this carefully designed vision-language bridging mechanism, the system can fully leverage the knowledge repository and reasoning capabilities of large language models to achieve deep understanding of complex driving scenarios and intelligent decision making based on natural language instructions. The high-level semantic representations output by the large language model are passed to the subsequent physics-constrained action generation module, where they are converted through waypoint decoders into executable trajectories and low-level control commands that satisfy vehicle dynamics constraints, realizing complete mapping from semantic understanding to physical execution.

### 3.3. Physics-Constrained Action Generation

The physical-constrained action generation module serves as the execution terminal of the entire VLA system, responsible for converting high-level semantic hidden states output by the large language model into executable trajectories and control commands that satisfy vehicle dynamics constraints. The core innovation of this module lies in introducing a cascaded architecture of GRU temporal state modeling [45] and bicycle model dynamics [49], ensuring that generated trajectories are not only semantically reasonable but, more importantly, physically feasible and safe. Unlike traditional methods that directly output trajectory coordinates, this module achieves reliable mapping from semantic understanding to physical execution by modeling temporal dependencies and physical constraints of vehicle motion.

The system employs a GRU recurrent neural network to model the temporal motion states of the vehicle, effectively capturing the influence of historical state information on future trajectories. The GRU unit takes the vehicle’s three-dimensional state vector [x,y,θ] as input, where *x* and *y* represent the vehicle’s position in the BEV coordinate system, and θ represents the vehicle’s heading angle. Compared to traditional methods that only consider positional information, introducing the heading angle enables the system to more accurately model vehicle motion trends and steering behaviors. The GRU’s hidden state is initialized by the output features of the large language model, ensuring that the temporal modeling process can fully utilize semantic information from language instructions and visual scenes. At each time step, the GRU predicts control variables based on the current state and hidden state, including vehicle velocity *v* and steering angle δ, which are subsequently passed to the physical dynamics module for constraint processing.

To ensure the physical feasibility of generated trajectories, the system integrates vehicle dynamics constraints based on the bicycle model [49]. The bicycle model, as a classical simplified model of vehicle kinematics, can accurately describe vehicle steering and driving behaviors while maintaining computational efficiency. The model adopts fixed parameters as specified in the LMDrive dataset: a 3.1 m wheelbase corresponding to standard vehicle configurations in the CARLA [50] simulation environment, and a 0.1 s discrete time step, ensuring consistency with the dataset configuration and fair comparison with other methods. The system performs state updates through kinematic equations of the bicycle model: new positions are calculated through current velocity, heading angle, and time step, while new heading angles are updated based on velocity, steering angle, and wheelbase parameters. To maintain numerical stability of angles, the system performs [−π,π] range normalization of heading angles after each step update.

To achieve balanced optimization of trajectory quality, physical feasibility, and safety, the system employs a multi-objective loss function that combines trajectory prediction, safety constraints, and termination prediction:

(4) $$L_{action}=\lambda_{traj}L_{traj}+\lambda_{end}L_{end}+\lambda_{safety}L_{safety}$$

where the trajectory loss is

(5) $$L_{traj}=\sum_{i=1}^{N}\left|\right|w_{i}-{\hat{w}}_{i}{\left|\right|}_{1}$$

where $w_{i}$ and ${\hat{w}}_{i}$ represent the ground truth and predicted waypoints, respectively, for measuring the L1 distance between predicted and ground truth waypoints. The safety loss is

(6) $$L_{\mathrm{safety}}=\sum_{i=1}^{N}\sum_{j=1}^{M_{i}}{\left(\max\;\left(0,\;d_{\mathrm{safe}}-{∥w_{i}-a_{ji}∥}_{2}\right)\right)}^{2}$$

which penalizes trajectories that are too close to other traffic participants, where $a_{ji}$ denotes the position of actor *j* at time step *i* ($j=1,\ldots ,M_{i}$), and $d_{\mathrm{safe}}$ is the safety-distance threshold. And the termination loss is

(7) $$L_{end}=-\sum_{i=1}^{N}\left[e_{i}\log\sigma \left({\hat{e}}_{i}\right)+\left(1-e_{i}\right)\log\left(1-\sigma \left({\hat{e}}_{i}\right)\right)\right]$$

for predicting when the vehicle should complete its trajectory sequence with $e_{i}\in \left\{0,1\right\}$ denoting termination labels. The trajectory termination prediction loss is designed to predict when the vehicle should complete its current trajectory sequence, enabling the system to determine appropriate stopping points or transition moments for different driving maneuvers.

Through this cascaded architecture design of GRU temporal modeling and the bicycle model, the system achieves reliable conversion from high-level semantic decisions to low-level control commands. The generated 5 waypoints not only respond to language instructions and visual scene requirements at the semantic level but also satisfy vehicle dynamics constraints and safety requirements at the physical level. The waypoints are subsequently converted to vehicle control commands (steering, throttle, brake) through PID controllers for deployment in the CARLA simulation environment. This physics-aware action generation mechanism effectively addresses the key deficiency of traditional VLA models in trajectory physical feasibility, laying an important foundation for the practical deployment of language-conditioned autonomous driving. The complete physics-constrained action generation process is summarized in Algorithm 2.
**Algorithm 2** Physics-Constrained Action Generation **Require:**LLM hidden states R; initial state $s_{0}=\left[x_{0},y_{0},\theta_{0}\right]$ **Ensure :**control commands
 
// Initialize GRU temporal state modeling

**_1_**
h←GRU_InitialHidden(R);

**_2_** state $\leftarrow s_{0}$;   [x,y,θ]←state;

**_3_** waypoints ←[];
 
// Iterative trajectory generation with bicycle model

**_4_**
**for**
*step = 1 to 5*
**do**;


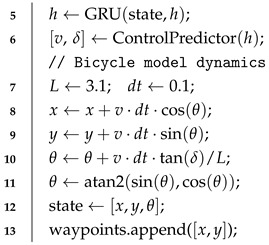

 // Convert waypoints to control commands

**_14_**
[steer,throttle,brake]←PIDControl(waypoints,v);

## 4. Experiments

### 4.1. Datasets

We conduct experiments on the LMDrive dataset [12], a large-scale multimodal dataset specifically designed for language-conditioned autonomous driving research. The dataset comprises approximately 64,000 instruction-sensor-control data clips collected in the CARLA simulator. Each clip contains one navigation instruction, several notice instructions, a sequence of multimodal multi-view sensor data, and control signals, with durations ranging from 2 to 20 s.

The dataset was collected using a rule-based expert agent across 8 towns in CARLA 0.9.10.1, encompassing diverse weather conditions and routes. The sensor configuration includes multi-view cameras (400 × 1200 resolution, which can be split into left, center, right, and rear views) and LiDAR point clouds (covering 180 degrees of horizontal field of view). Data is collected at a frequency of approximately 10Hz, ensuring rich temporal information.

### 4.2. Implementation Details

In the Env Perception Module pre-training stage, we use 8 RTX A6000 GPUs to train for 25 epochs. The multi-view input size is set to 3 × 128 × 128, integrating multi-view cameras, LiDAR, and map information. Training uses the AdamW optimizer with a learning rate of 0.001, backbone learning rate of 0.0004, and weight decay of 0.05. The learning rate scheduling adopts a cosine annealing strategy with 5 epochs of warmup. The scene understanding loss function weights are set to $\lambda_{prob}=0.4$, $\lambda_{loc}=\lambda_{box}=\lambda_{ori}=0.2$, with reference to LMDrive [12] and InterFuser [30].

In the Driving VLA module training stage, we use 8 RTX A6000 GPUs (NVIDIA Corporation, Santa Clara, CA, USA) to train for 10 epochs. We adopt LLaVA-v1.5-7B as the base large language models, with the maximum text length set to 64. Training uses linear warmup cosine learning rate scheduling with an initial learning rate of 0.0002 and minimum learning rate of 0.00002, and integrates a GRU encoder to process temporal information. The action generation loss function weights are set to $\lambda_{traj}=1.0$, $\lambda_{end}=0.2$, $\lambda_{safety}=0.1$.

The evaluation stage runs on a local RTX3090 single GPU, using the CARLA 0.9.10.1 simulator for closed-loop testing.

### 4.3. Evaluation Metrics

We adopt the CARLA Leaderboard [51] evaluation protocol to assess the driving proficiency of our VLA autonomous driving system. The evaluation quantifies driving performance across multiple dimensions through a comprehensive set of metrics.

Route Completion (RC) measures the percentage of route distance successfully completed by the agent, $R_{i}$, ranging from 0 to 100.

The Infraction Score (IS) aggregates all infractions through the formula:

$$P_{i}=\frac{1}{1+\sum_{j}c_{j}\times \#{\mathrm{infractions}}_{j}}$$

where agents begin with a base score of 1.0, $c_{j}$ represents the penalty coefficient for infraction type *j*, and $\#{\mathrm{infractions}}_{j}$ is the number of infractions of type *j*.

The Driving Score (DS) serves as the primary evaluation metric, defined as the product of route completion rate $R_{i}$ and infraction penalty $P_{i}$: $\mathrm{DS}=R_{i}\times P_{i}$, where $R_{i}$ represents the completion percentage of the *i*-th route and $P_{i}$ denotes the corresponding infraction penalty coefficient, with a maximum value of 100.

The evaluation system monitors various types of infractions with corresponding penalty coefficients: collisions with pedestrians (1.0), collisions with other vehicles (0.70), collisions with static objects (0.60), running red lights (0.40), violating stop signs (0.25), and off-road driving (proportional penalty).

Upon completion of all test routes, the system calculates global values for each metric using arithmetic averaging across all routes. The global driving score serves as the primary benchmark for inter-system performance comparison.

### 4.4. Results and Analysis

#### 4.4.1. Overall Performance Comparison

We conducted a comprehensive quantitative evaluation on the LangAuto benchmark to assess the effectiveness of VLA-MP. Table 1 reports the comparison results against existing baselines under three evaluation settings: LangAuto (routes > 500 m), LangAuto-Short (150–500 m routes), and LangAuto-Tiny (<150 m routes).

Our model, built upon LLaVA-7B, achieves the best driving scores across all three settings, with DS values of 44.3, 63.5, and 78.4, respectively. In terms of the route completion rate, the method also reaches high levels of 49.6, 71.1, and 82.3, indicating consistent performance across different route lengths and levels of complexity. Regarding safety, the proposed approach yields infraction scores of 0.89, 0.90, and 0.95, demonstrating reduced violation rates. These results reflect the contribution of the physics-constrained action generation module and the GRU-bicycle dynamics cascade, which together help ensure the feasibility and safety of the generated trajectories.

Performance on shorter routes (LangAuto-Tiny) is notably higher, suggesting that the framework is reliable in relatively simple driving tasks. As route length and complexity increase, performance decreases but remains competitive, confirming the robustness and practicality of the approach. Overall, the results validate the effectiveness of the end-to-end vision–language–action architecture in handling language-conditioned driving scenarios, and highlight the potential of integrating large language models into autonomous driving systems. The performance variations among baselines reflect differences in model capacity (e.g., DSDrive’s [14] 1B vs. 7B parameters), training strategies, hierarchical BEV feature extraction, and the lack of physics-constrained trajectory generation in existing VLA methods.

#### 4.4.2. Qualitative Visualization Results

This section presents specific driving scenarios to deeply analyze the decision-making process and execution performance of the VLA autonomous driving framework in complex environments. To intuitively demonstrate the complete perception-decision-execution pipeline of the system, we designed a customized HUD (Head-Up Display) interface. This interface integrates multiple information sources, including multi-view camera inputs (left view, focus view, right view) providing 360-degree environmental perception, the main driving perspective displaying front road conditions, a system status panel showing real-time time, the frame count and vehicle speed, a vehicle control panel displaying throttle, steering, and brake values with progress bars, and a navigation information panel presenting planned waypoint coordinates and current language instructions.

**Figure 5:**
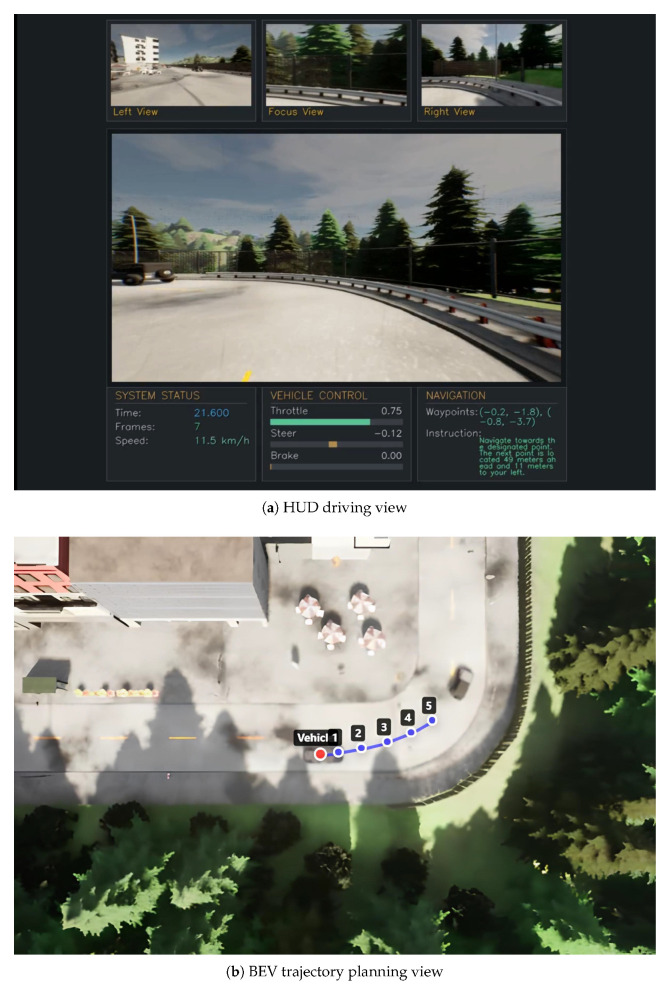
Spatial navigation scenario demonstration. (**a**) HUD interface displaying multi-view camera inputs, vehicle controls, and navigation information guided by language instructions. (**b**) BEV trajectory planning view with the numbered waypoints (1–5), indicating the planned path.

As shown in Figure 5, when the system receives the instruction “Get to the point, the next one’s just 49 m ahead and 11 m to your left”, it demonstrates the understanding and execution capability for specific spatial distance instructions. The system accurately parses the spatial position information, with the vehicle traveling at 8.7 km/h and a steering value of −0.05 indicating execution of a slight left turn action. The corresponding BEV clearly shows the trajectory planning generated by the system, marked by numbered waypoint sequences 1–5 indicating the target path, validating that our vision-language bridge module can effectively convert spatial descriptions in natural language into precise trajectory planning.

**Figure 6:**
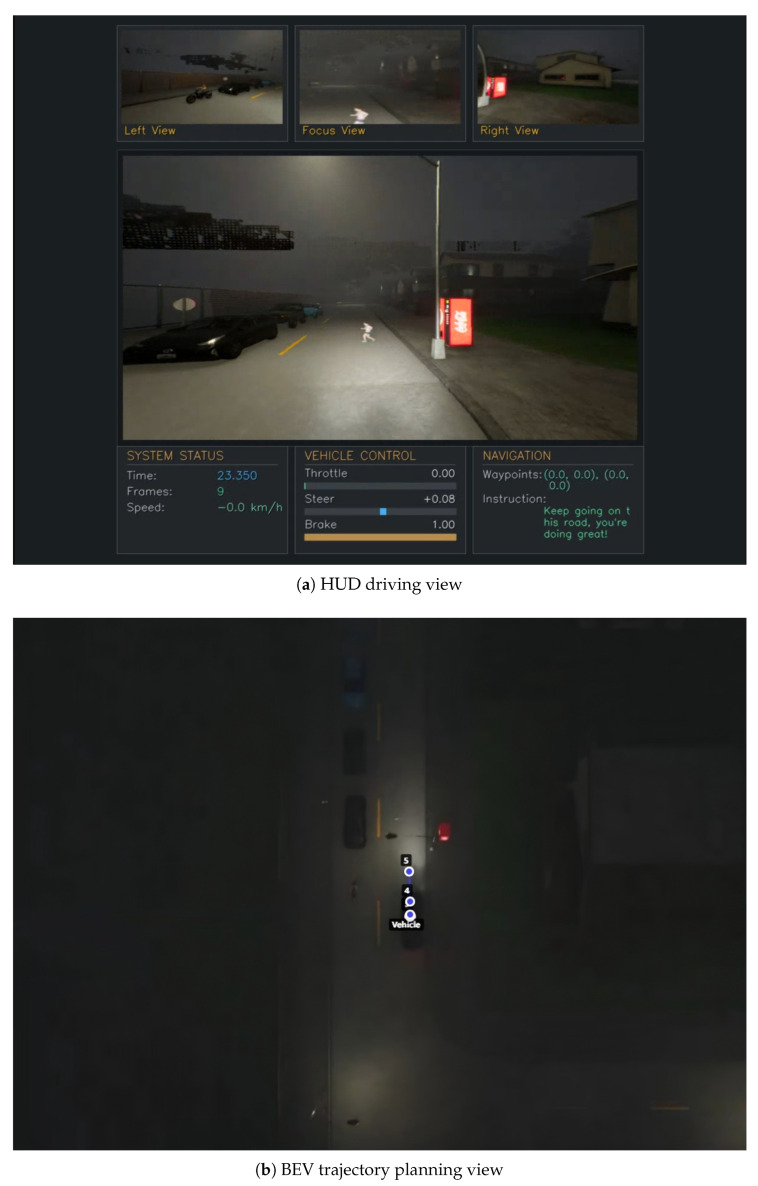
Safety-aware emergency stop scenario. (**a**) HUD interface showing the emergency stop operation with a speed of 0.0 km/h and braking upon detecting pedestrians in a nighttime environment. (**b**) BEV view showing overlapping waypoints at the current position, indicating the vehicle’s decision to wait until the pedestrian pass.

The nighttime scenario shown in Figure 6 highlights the system’s safety perception and emergency decision-making capabilities. Facing the instruction “Preserve your current trajectory until the forthcoming intersection”, the system detects pedestrians ahead in the nighttime urban environment and immediately executes safety stop operations: speed reduces to 0.0 km/h with the brake value reaching 1.00. From the navigation panel in the interface and the bird’s-eye view, it can be observed that the first three waypoints planned by the system almost overlap at the current position, indicating that the vehicle chooses to wait in place until predicting when the pedestrian passes before continuing forward. This behavior demonstrates the system’s comprehensive decision-making capability, showing that while executing language instructions, it can simultaneously perceive environmental changes in real time and prioritize driving safety, reflecting the effective integration of multimodal perception and intelligent decision making.

#### 4.4.3. Dynamic Video Demonstrations

In addition to the HUD interface design and static scene analysis, we provide three video demonstrations (see Supplementary Materials) to more intuitively showcase the performance of the proposed VLA framework during continuous driving processes. Unlike static images, these videos can comprehensively present the system’s perception–understanding-execution process across temporal sequences, thereby highlighting the model’s adaptability and reliability in dynamic environments.

Videos S1 and S2 correspond to the scenarios shown in Figure 5 and Figure 6, respectively. These two videos complement the static visualization results by demonstrating the system’s understanding of language instructions, perception of environmental elements, and subsequent action execution processes in the same environments. Through dynamic demonstrations, the gradual completion of behaviors such as steering, acceleration, or collision avoidance by the vehicle in interactive scenarios can be observed more clearly.

Video S3 presents a longer driving segment (approximately 50 s) that focuses on demonstrating the system’s long-term sequential decision-making and execution capabilities in more complex scenarios. Unlike the previous two short-term tasks, this case reflects the model’s consistency in multi-step task planning, maintaining coherent goal-oriented behavior in dynamic traffic environments, further validating the stability and effectiveness of the proposed method over extended time sequences.

#### 4.4.4. Ablation Studies

To validate the contribution of different components in our VLA autonomous driving framework, we conduct comprehensive ablation studies on the LangAuto-Tiny benchmark. Table 2 presents the performance comparison when removing key components from our complete system.

Eliminating the projector module causes a 10.9-point drop in driving score, underscoring the necessity of robust vision-language alignment. The projector’s cross-attention mechanism selectively extracts driving-relevant information from BEV features, effectively bridging the gap between visual perception and language reasoning.

Without physical constraints, the driving score decreases by 20.4 points. This result confirms that directly predicting coordinates from a language model-without accounting for vehicle dynamics-produces infeasible trajectories that cannot be executed safely. By cascading a GRU with a bicycle model, the system enforces temporal consistency and kinematic feasibility, both of which are essential for reliable autonomous driving.

The absence of environmental pre-training leads to the most severe performance collapse, with the driving score plunging by 36.1 points and the infraction score falling from 0.95 to 0.69. This demonstrates the foundational role of structured scene understanding provided by the pre-trained BEV perception module. Without a solid multimodal environmental representation, the system struggles to interpret complex driving scenarios and fails to generate appropriate responses to language instructions.

These ablation studies confirm that each component plays a vital role in the overall system performance. Environmental pre-training provides the foundation for structured scene understanding, physical constraints guarantee safety and feasibility, and the projector enables efficient multimodal fusion. Together, they validate the architectural choices of our VLA framework.

#### 4.4.5. Computational Efficiency Analysis

To evaluate the practical deployment potential of our VLA autonomous driving framework, we conduct a comprehensive computational efficiency analysis on a single RTX 3090 GPU. Table 3 summarizes the performance metrics of our system during inference.

The experimental results show that our framework achieves a total processing time of 125.04 ms per frame, corresponding to a processing rate of 8.0 FPS on the RTX 3090 platform. Among the core computational components, the visual processing module takes 43.78 ms, the LLM inference module requires 20.91 ms, and the physics control module only needs 0.25 ms. The remaining time is primarily attributed to CARLA simulation environment communication overhead and system-level data processing. Visual processing and LLM inference serve as the two main computational bottlenecks, fully demonstrating the computational complexity brought by the deep integration of high-dimensional BEV perception and large language model inference.

In performance comparison with existing VLA methods, our framework demonstrates significant computational efficiency advantages. AutoVLA [7] operates at approximately 1 FPS, while FastDriveVLA [53] achieves about 4.86 FPS. In contrast, our method reaches 8.0 FPS, showing excellent performance among current VLA methods and proving the effectiveness of our architectural design and optimization efforts.

However, when compared with traditional end-to-end autonomous driving methods evaluated in CARLA closed-loop environments, we find there is still significant room for optimization. Traditional methods such as TransFuser [54] have a runtime of 44 ms, and TF++ [40] has a runtime of 50 ms, both significantly lower than our 125.04 ms. This performance gap indicates that while VLA methods have unique advantages in providing superior interpretability and complex reasoning capabilities, they still require further technological breakthroughs in pure computational efficiency to match traditional end-to-end systems.

From a system resource perspective, our framework shows a peak GPU memory usage of 13.7 GB during inference, which is well within the capacity of modern high-end consumer GPUs, providing good hardware compatibility for practical deployment. The model contains a total of 6.9 billion parameters, with the vast majority coming from the large language model backbone, enabling the system to possess strong language understanding, logical reasoning, and scene analysis capabilities.

From a practical deployment perspective, the current experimental results point to clear directions for future optimization work. Model quantization techniques can effectively reduce parameter precision to lower computational complexity, knowledge distillation methods can compress large model knowledge into smaller networks, and specialized hardware accelerators can be optimized for specific computational patterns. The comprehensive application of these techniques is expected to significantly improve inference efficiency while maintaining model capabilities, thereby narrowing the performance gap with traditional end-to-end methods and laying a solid foundation for the widespread application of VLA methods in real-world autonomous driving systems.

## 5. Conclusions

This paper proposes VLA-MP, a unified end-to-end vision-language-action framework for autonomous driving. The framework consists of three core modules: a multimodal environmental perception module that fuses heterogeneous sensor data into structured BEV representations and extracts hierarchical features, a vision-language bridge and large language action module that enables cross-modal semantic alignment and high-level decision generation, and a physics-constrained action generation module that ensures both semantic reasonableness and physical feasibility of trajectories through a GRU-bicycle model cascade. Through a three-stage progressive training strategy, the framework achieves seamless mapping from sensor observations and natural language instructions to executable control commands.

Comprehensive experiments on the LMDrive dataset validate the effectiveness of our approach. Quantitative results demonstrate that our method achieves the best driving performance across all evaluation settings, showing consistent performance across different route complexities. Qualitative visualization results through our designed HUD interface showcase the system’s capability in spatial navigation and safety decision scenarios, accurately understanding language instructions and generating reasonable driving behaviors. Dynamic video demonstrations further confirm the system’s stability and adaptability during continuous driving processes. Ablation studies verify the importance of each proposed component, with environmental pre-training providing the system foundation, physical constraints ensuring trajectory safety, and projectors enabling effective multimodal fusion. Computational efficiency analysis indicates that the system achieves acceptable inference performance, providing clear directions for future optimization.

Despite achieving promising experimental results, our system still has several limitations. First, the current inference speed cannot meet the typical frequency requirements for real-time autonomous driving, primarily constrained by the computational complexity of large language models. The computational heaviness of LLMs presents deployment challenges in terms of processing requirements and operational costs. Second, our evaluation reveals challenges in handling complex and lengthy scenarios and routes. The system’s performance degrades on long-distance complex routes, indicating difficulties in maintaining coherent decision making over extended temporal sequences and processing of lengthy contextual information. Third, the system’s operation with complex and implicit user requests remains constrained. Handling ambiguous instructions, multi-step commands, or contextual references that require deeper understanding of unstated intentions presents ongoing challenges for robust language understanding in driving scenarios. Additionally, our evaluation is mainly conducted in the CARLA simulation environment, and real-world generalization capabilities and robustness require further validation. Real-world deployment would face additional challenges including sensor noise and calibration drift, dynamic weather conditions affecting perception reliability, and unexpected traffic scenarios not covered in simulation datasets.

In future work, we plan to improve computational efficiency through model compression, knowledge distillation, and specialized hardware optimization techniques to meet real-time deployment requirements. We will validate the system’s generalization capabilities on other datasets and more diverse driving scenarios. We also aim to investigate more sophisticated language understanding capabilities, including multi-turn dialogue, contextual reasoning, and instruction disambiguation, to achieve more natural human-machine interactive autonomous driving experiences.

## Figures and Tables

**Table 1:** Overall performance comparison on the LangAuto benchmark under three evaluation settings: LangAuto, LangAuto-Short, and LangAuto-Tiny. Higher values indicate better performance (↑). Best results are highlighted in bold.

LLM Backbone	LangAuto			LangAuto-Short			LangAuto-Tiny		
	DS ↑	RC ↑	IS ↑	DS ↑	RC ↑	IS ↑	DS ↑	RC ↑	IS ↑
Random Init.	10.7	16.2	0.63	14.2	20.1	0.72	20.1	24.7	0.75
LMdrive (LLaMA-7B) [12]	31.3	37.1	0.82	42.8	49.1	0.87	52.5	57.8	0.91
LMdrive (LLaVA-7B)	36.2	46.5	0.81	50.6	60.0	0.84	66.5	77.9	0.85
AD-H (LLaVA-7B) [52]	44.0	**53.2**	0.83	56.1	68.0	0.78	77.5	**85.1**	0.91
AD-H (Mipha-3B)	41.1	48.5	0.86	54.3	61.8	0.86	68.0	74.4	0.87
BEVdriver (Llama3.1-8B-I) [6]	33.1	40.7	0.83	60.9	65.8	**0.92**	66.0	69.9	0.90
DSDrive (LLaMA-1B) [14]	29.5	39.3	0.77	62.0	**76.1**	0.81	60.6	72.5	0.84
Ours VLA-MP (LLaVA-7B)	**44.3**	49.6	**0.89**	**63.5**	71.1	0.90	**78.4**	82.3	**0.95**

**Table 2:** Ablation study results on LangAuto-Tiny benchmark. All experiments use LLaVA-7B as the backbone. Higher values indicate better performance (↑). Best results are highlighted in bold.

Module Design	DS ↑	RC ↑	IS ↑
Ours(VLA-MP)	**78.4**	**82.3**	**0.95**
w/o Projector	67.5	75.0	0.91
w/o Physical-Constrained	58.0	64.0	0.92
w/o Env Pre-training	42.3	61.1	0.69

**Table 3:** Computational efficiency analysis of the proposed VLA framework on RTX 3090 GPU.

Metric	Value	Unit
Total Time	125.04	ms
Visual Processing	43.78	ms
LLM Inference	20.91	ms
Physics Control	0.25	ms
FPS	8.0	frames/s
Peak GPU Memory	13.7	GB
Model Parameters	6.9	B
Hardware Platform	RTX 3090	-

## Data Availability

The experiments in this study were conducted using the publicly available LMDrive dataset, which can be accessed at https://huggingface.co/datasets/OpenDILabCommunity/LMDrive (accessed on 20 August 2025). The CARLA simulator (version 0.9.10.1) used for evaluation is open source and available at https://carla.org/ (accessed on 20 August 2025).

## Supplementary Materials

The following supporting information can be downloaded at: https://www.mdpi.com/article/10.3390/s25196163/s1, Video S1: Spatial navigation scenario demonstration; Video S2: Safety-aware emergency stop scenario demonstration; Video S3: Long-sequence driving scenario (50 s).

## Abbreviations

The following abbreviations are used in this manuscript:

VLA	Vision–Language–Action
BEV	Bird’s Eye View
LLM	Large Language Model
GRU	Gated Recurrent Unit
FPS	Frames Per Second
DS	Driving Score
RC	Route Completion
IS	Infraction Score
HUD	Head-Up Display
